# A male-specific association between *AGTR1* hypermethylation and coronary heart disease

**DOI:** 10.17305/bjbms.2019.4321

**Published:** 2020-02

**Authors:** Xiaojing Li, Nan Wu, Huihui Ji, Yi Huang, Haochang Hu, Jiyi Li, Siyu Mi, Shiwei Duan, Xiaomin Chen

**Affiliations:** 1 Key Laboratory of Ningbo First Hospital and Cardiovascular Center of Ningbo First Hospital, Ningbo University, Ningbo, China; 2 Medical Genetics Center, School of Medicine, Ningbo University, Ningbo, China

**Keywords:** Coronary heart disease, CHD, *AGTR1*, DNA methylation, males, angiotensin II receptor type 1, HepG2 cells, 5-aza-2’-deoxycytidine

## Abstract

The *AGTR1* gene encodes angiotensin II receptor type 1, which is involved in cardiovascular diseases such as coronary heart disease (CHD). In the current study, we analyzed *AGTR1* promoter methylation level in a Han Chinese population by SYBR green-based quantitative methylation-specific PCR (qMSP). We collected blood samples from 761 CHD patients and 398 non-CHD controls at the Ningbo First Hospital. A data mining analysis was also performed to explore the association between *AGTR1* methylation and *AGTR1* gene expression, using datasets from the cBioPortal for Cancer Genomics and the Gene Expression Omnibus (GEO) database. Our results showed a significantly higher percentage of methylated reference (PMR) of *AGTR1* in male CHD patients compared with male non-CHD controls (median PMR: 2.12% vs. 0.59%, *p* = 0.037). The data mining analysis showed that *AGTR1* expression was significantly increased in human hepatoma HepG2 cells treated with the demethylation agent 5-aza-2’-deoxycytidine (fold = 3.12, *p* = 0.009). Further data mining analysis using the cholangiocarcinoma (TCGA, PanCancer Atlas) data indicated an inverse association between *AGTR1* methylation and *AGTR1* expression (r = -0.595, *p* = 1.29E-04). Overall, our results suggest that *AGTR1* methylation is involved in the regulation of *AGTR1* gene expression and that *AGTR1* hypermethylation is associated with CHD in males. These findings may provide new clues about the pathogenesis of CHD.

## INTRODUCTION

Coronary heart disease (CHD) is a type of cardiovascular disease, which remains a significant public health problem globally [1,2]. CHD is a complex disease influenced by both environmental and genetic factors. Previous studies suggested that epigenetic modifications are involved in the pathophysiology of CHD [3,4].

An important epigenetic mechanism of gene expression regulation is DNA methylation, which represents the methylation of cytosine to 5-methylcytosine, primarily at the CpG sites [5]. Recent studies showed that the methylation level of *FOXP3*, *BAX*, and *PON1* is significantly associated with CHD [6-8]. Interestingly, *CDKN2B* promoter methylation was significantly associated with the risk of CHD in women but not in men [9], suggesting its sex-dependent effects in the pathogenesis of CHD.

Angiotensin II is a potent vasopressor hormone that plays a vital role in blood pressure regulation and is associated with the pathogenesis of CHD [10]. Angiotensin II mainly acts through angiotensin II receptor type 1 (AGTR1) and angiotensin II receptor type 2 (AGTR2), which mediate its cardiovascular effects [11,12]. AGTR1 is a member of the G-protein-coupled receptor superfamily and is encoded by a gene on chromosome 3q [12]. Previous genetic studies found that *AGTR1* variants are associated with essential hypertension in Polish and Finnish populations [13,14]. Moreover, *AGTR1* rs3772622 polymorphism increases the risk of CHD in Northern Han Chinese patients with non-alcoholic fatty liver disease (NAFLD) [15]. On the other hand, another study on Chinese patients showed no correlation between CHD and 8 *AGTR1* haplotypes with a frequency greater than 3% [16]. Similarly, in our previous study, we did not find an association between four CpG-SNPs on the *AGTR1* promoter and CHD in a Han Chinese population [17].

Aberrant *AGTR1* methylation is associated with multiple diseases. *AGTR1* hypermethylation increases the risk of oral cancer [18] and non-small-cell lung carcinoma [19]. A previous study found a significantly lower *AGTR1* methylation in hypertensive patients than in healthy controls [20].

The relationship between *AGTR1* methylation and CHD is still not clear. Therefore, in this study, we aimed to explore the association of *AGTR1* promoter methylation with CHD in a Han Chinese population.

## MATERIALS AND METHODS

### Patients

We collected blood samples from 761 CHD patients (501 men and 260 women, median age: 62 years) and 398 non-CHD controls (220 men and 178 women, median age: 60 years) at the Ningbo First Hospital. CHD patients were diagnosed with coronary artery stenosis greater than 50% in one or more of the major coronary arteries. The control group consisted of inpatients with coronary artery stenosis less than 50% in the major coronary arteries and without any atherosclerotic vascular disease. Standardized coronary angiography with Seldinger’s technique was performed in all patients [21,22]. The results of the angiography were independently evaluated by at least two cardiologists. None of the control inpatients had histories of congenital heart disease, cardiomyopathy, and severe liver or kidney disease. The Institutional Review Board of Ningbo First Hospital and Ningbo University approved the study (NBU-CHD-20180305). All participants provided written informed consent form.

### SYBR green-based quantitative methylation-specific PCR (qMSP)

We extracted genomic DNA from peripheral blood samples as previously described [23]. The details of bisulfite conversion and qMSP procedures are available from our previous studies [24,25]. To measure the methylation of AGTR1 promoter we calculated the percentage of methylated reference (PMR) [24,25]. The primer sequences used in qMSP were forward 5’-GGAGGAGGAGGGAATGTAA-3’ and reverse 5’-CCTATCACTCGCTACTACCT-3’.

### Data mining analysis

A data mining analysis was performed to explore the association between *AGTR1* methylation and *AGTR1* gene expression. We obtained the cholangiocarcinoma dataset (TCGA, PanCancer Atlas) from the cBioPortal for Cancer Genomics (http://www.cbioportal.org) [26]; *AGTR1* methylation and *AGTR1* gene expression were determined with Infinium HumanMethylation450 BeadChip and RNAseq, respectively. In addition, we used the data for human hepatoma HepG2 cells from the Gene Expression Omnibus (GEO) database (GSE5230).

### Statistical analysis

PASW statistics 18.0 software (SPSS Inc., Somers, NY, USA) was used for statistical analysis. We compared continuous variables between the two groups by a t-test or a non-parametric test. A univariate linear regression analysis was used to determine the individual risk factors, then a multivariate regression analysis was performed to explore the association between these factors and CHD. A Spearman’s correlation test was used to assess the association between *AGTR1* methylation and each of the 8 metabolic parameters, as follows low-density lipoprotein (LDL), total cholesterol (TC), triglyceride (TG), apolipoprotein A1 (ApoA1), apolipoprotein B (ApoB), apolipoprotein E (ApoE), lipoprotein A [Lp(a)], and high-sensitivity C-reactive protein (hs-CRP). A two-tailed *p* < 0.05 was considered to be significant.

## RESULTS

**FIGURE 1:**
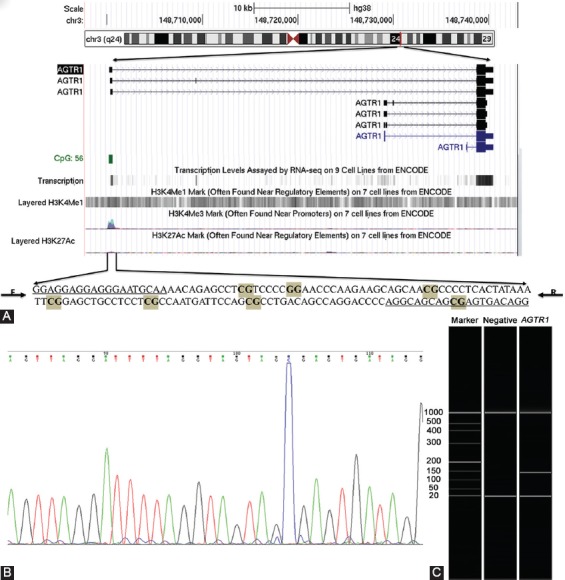
DNA fragment tested in the *AGTR1* methylation assay. (A) The genomic position of the amplified fragment in *AGTR1x* gene CpG island; F and R stand for forward and reverse primer, respectively. (B) DNA sequencing indicated a good bisulfite conversion. (C) Gel electrophoresis showed the correct size of the amplified DNA fragment (142 bp).

In this study, we included 761 CHD patients (mean age: 58.52 ± 9.52) and 398 controls [mean age: 61.41 ± 9.29] (Table 1). As shown in Figure 1, the selected region for the *AGTR1* methylation assay is located in a transcription factor-binding site. The methylation level of an *AGTR1* CpG was measured, representing a 142-bp amplified fragment in the CpG island of *AGTR1* (Figure 1).

**TABLE 1:**
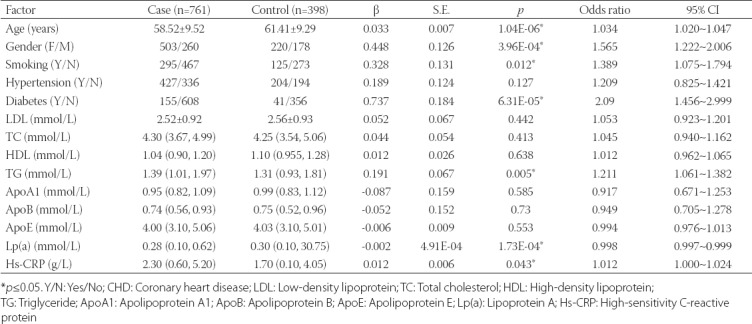
Univariate logistic regression analysis of environmental factors that may influence CHD in the Han Chinese population

Demographic characteristics and laboratory parameters, including LDL, TC, TG, ApoA1, ApoB, ApoE, Lp(a), and hs-CRP are shown in Table 1. Our univariate regression analyses showed that age, male sex, cigarette smoking, diabetes, TG, and hs-CRP were the risk factors of CHD (all *p* < 0.05, Table 1). A multivariate regression analysis showed that 4 factors (age, male sex, diabetes, and TG) were the risk factors and Lp(a) was the protective factor of CHD (all *p* < 0.05, Table 2). However, *AGTR1* methylation was not associated with CHD in the subgroup analyses by diabetic status (*p* > 0.05, data not shown). We found no significant relationships between *AGTR1* methylation and clinical indexes, including age, TG, and Lp(a) (*p* > 0.05, data not shown).

**TABLE 2:**
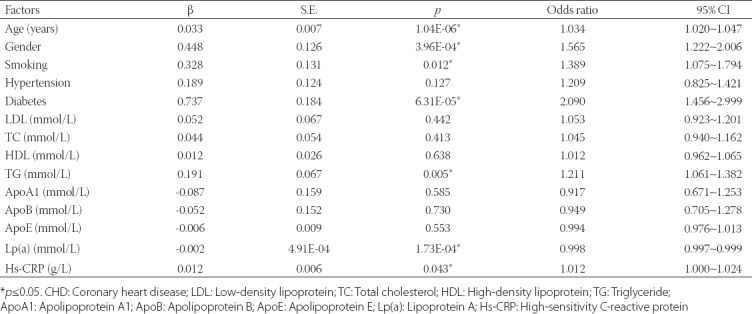
Multivariate logistic regression analysis of environmental factors that may influence CHD in the Han Chinese population

**FIGURE 2:**
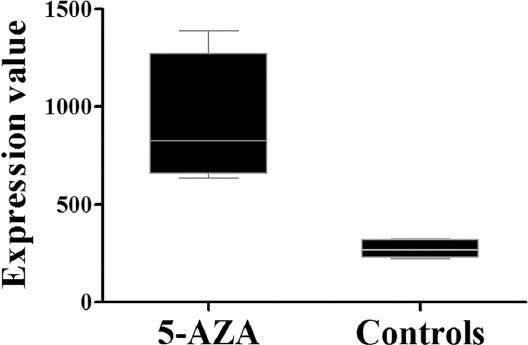
The data mining analysis showed that the demethylation agent 5-aza-2’-deoxycytidine significantly increased *AGTR1* gene expression in treated human hepatoma HepG2 cells vs. controls (fold change = 3.12, p = 0.009).

**FIGURE 3:**
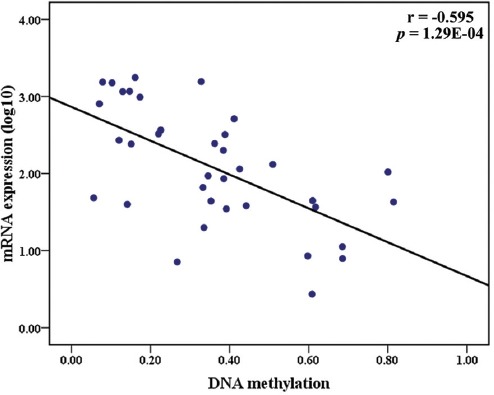
The Spearman’s rank correlation test using the cholangiocarcinoma (TCGA, PanCancer Atlas) data indicated an inverse association between *AGTR1* methylation and *AGTR1* expression (r = -0.595, p = 1.29E-04). The x-axis shows *AGTR1* methylation levels. The y-axis shows log-transformed *AGTR1* gene expression values.

*AGTR1* methylation was shown to be significantly higher in male CHD patients than in male controls (mean PMR: 2.12 vs. 0.59, *p* = 0.037, Table 3). Furthermore, GEO data analysis showed a significantly decreased *AGTR1* expression in HepG2 cells treated with a demethylation agent (5-aza-2’-deoxycytidine [5-AZA], fold = 3.12, *p* = 0.009, Figure 2). Further bioinformatics analysis using the cholangiocarcinoma (TCGA, PanCancer Atlas) dataset indicated a significant inverse association between *AGTR1* methylation and *AGTR1* gene expression (r = -0.595, *p* = 1.29E-04, Figure 3). All the above results suggested a pivotal role of *AGTR1* methylation in the regulation of *AGTR1* gene expression.

**TABLE 3:**
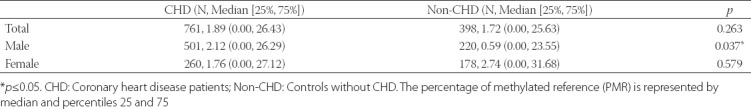
A comparison of *AGTR1* methylation between CHD and non-CHD groups in the Han Chinese population

In our previous study, we found no association of CHD with CpG-SNP rs275653 on the *AGTR1* promoter [17]. Here, we performed an interaction test between rs275653 and *AGTR1* methylation. We did not observe a significant interaction between the rs275653 SNP and methylation level (data not shown).

## DISCUSSION

In the present study, we explored the association between *AGTR1* methylation and CHD. We found four risk factors (age, male sex, diabetes, and TG) and one protective factor [Lp(a)] of CHD. Although there was no association of *AGTR1* methylation with CHD in the total sample, we found elevated *AGTR1* methylation in male CHD patients compared with male non-CHD controls. Moreover, the data mining analyses showed that a demethylation agent could induce *AGTR1* expression and that *AGTR1* expression was inversely associated with *AGTR1* methylation. These results suggest that *AGTR1* methylation contributes to the risk of CHD in males through the regulation of *AGTR1* gene expression.

Sex differences exist in the incidence and development of CHD as well as in the frequency of CHD-related polymorphisms [27]. The incidence of CHD is higher in men than in women, in all age groups [28]. Men have a higher prevalence of ST-elevation myocardial infarction but are less likely to have heart failure compared with women [29]. The difference in the distribution of CHD between males and females is possibly due to different lifestyles, including smoking, alcohol consumption, high sodium diet, and physical activity [30-33].

A previous study showed that males with the deletion/deletion (D/D) angiotensin-I converting enzyme (*ACE*) gene polymorphism have a higher risk of premature myocardial infarction compared with women [34], suggesting a sex difference in the effect of *ACE* gene polymorphisms on CHD risk. In addition, sex hormones were suggested to regulate DNA methylation in cardiac calsequestrin 2 gene (*CASQ2*) promoter in mice [35]. A study investigating the association of *AGTR1* promoter methylation with the risk of essential hypertension showed that CpG1 hypomethylation in the *AGTR1* promoter is likely associated with the risk of essential hypertension, with significantly lower CpG1 methylation levels in males than in females [20]. In the current study, we showed that male sex was a risk factor of CHD, and we detected *AGTR1* hypermethylation only in male patients with CHD. Therefore, the sex differences in *AGTR1* methylation levels in CHD patients may reflect the differences in lifestyle and sex hormone levels. AGTR1 mediates the cardiovascular effects of angiotensin II [36], and *AGTR1* promoter hypermethylation suppresses the gene expression. Our results indicated that sex-dependent difference exist in CHD and that *AGTR1* hypermethylation increases the risk of CHD by regulating *AGTR1* gene expression.

Some studies showed that an elevated Lp(a) level is an independent risk factor for CHD [37,38]. High levels of Lp(a) are independently associated with CHD-induced recurrent heart failure in patients with chronic heart failure [39]. Interestingly, contrary to the previous findings, our results suggest that Lp(a) may be a protective factor for CHD. Therefore, the role of Lp(a) in CHD should be further explored in larger-sample studies.

Although we demonstrated an association between *AGTR1* methylation and CHD in males, the cause-effect relationship remains unclear. In addition, we measured *AGTR1* methylation only in peripheral blood samples and DNA methylation profiles may vary among different tissues. Further studies with longitudinal cohort design and that include different tissues should confirm our findings.

## CONCLUSION

Overall, our results suggest that *AGTR1* methylation is involved in the regulation of *AGTR1* gene expression and that *AGTR1* hypermethylation is associated with CHD in males. These findings may provide new clues about the pathogenesis of CHD.
